# Multiple intrasyntenic rearrangements and rapid speciation in voles

**DOI:** 10.1038/s41598-018-33300-6

**Published:** 2018-10-08

**Authors:** Svetlana A. Romanenko, Natalya A. Serdyukova, Polina L. Perelman, Vladimir A. Trifonov, Feodor N. Golenishchev, Nina Sh. Bulatova, Roscoe Stanyon, Alexander S. Graphodatsky

**Affiliations:** 1 Institute of Molecular and Cellular Biology, SB RAS, Novosibirsk, Russia; 2 Novosibirsk State University, Novosibirsk, Russia; 3 Zoological Institute, RAS, Saint-Petersburg, Russia; 4 A.N. Severtsov Institute of Ecology and Evolution, RAS, Moscow, Russia; 5 Department of Biology, Anthropology Laboratories, University of Florence, Florence, Italy

## Abstract

Remarkably stable genomic chromosome elements (evolutionary conserved segments or syntenies) are the basis of large-scale chromosome architecture in vertebrate species. However, these syntenic elements harbour evolutionary important changes through intrachromosomal rearrangements such as inversions and centromere repositioning. Here, using FISH with a set of 20 region-specific probes on a wide array of 28 species, we analyzed evolution of three conserved syntenic regions of the Arvicolinae ancestral karyotype. Inside these syntenies we uncovered multiple, previously cryptic intrachromosomal rearrangements. Although in each of the three conserved blocks we found inversions and centromere repositions, the blocks experienced different types of rearrangements. In two syntenies centromere repositioning predominated, while in the third region, paracentric inversions were more frequent, whereas pericentric inversions were not detected. We found that some of the intrachromosomal rearrangements, mainly paracentric inversions, were synapomorphic for whole arvicoline genera or tribes: genera *Alexandromys* and *Microtus*, tribes Ellobini and Myodini. We hypothesize that intrachromosomal rearrangements within conserved syntenic blocks are a major evolutionary force modulating genome architecture in species-rich and rapidly-evolving rodent taxa. Inversions and centromere repositioning may impact speciation and provide a potential link between genome evolution, speciation, and biogeography.

## Introduction

It has repeatedly been hypothesized that the number of intrachromosomal rearrangements is underestimated both by cytogenetic and bioinformatic methods. However, the real extent and contribution of intrachromosomal rearrangements to the formation of genomes remains unknown. Limited genome sequencing data do not yet make it possible to adequately test this hypothesis. To test the impact of intrachromosomal rearrangements we chosen to study an exceptional group of rodents from the Arvicolinae (Cricetidae). Arvicolinae is a unique subfamily with the most complex phylogenomic history of all rodents^1–4^, which makes it a useful model to study interconnection between genome evolution and species radiation. The subfamily is one of the youngest and the most species-rich group of mammals. Arvicolinae are undoubtedly monophyletic, but the composition and phylogenetic relationships between even clearly defined tribes remain highly controversial^2,3,5^. The most primitive forms unquestionably belong to the group appeared approximately 7 million years ago. Currently taxonomy of the Arvicolinae recognizes about 150 species of voles and lemmings distributed across the Holarctic^2^. Nearly half of the arvicoline species are attributed to a single *Microtus* genus of voles radiated about 2 million years ago (e.g.)^2,6,7^. The division of the *Microtus* onto separate genera (*Alexandromys*, *Blanfordimys*, *Microtus*, *Terricola*) is not yet universally recognized^2,4,5^. Up to now the genome of only one species – *M*. *ochrogaster* – was sequenced^8^ but not assembled to the chromosomal level impeding application of bioinformatic tools for the evaluation of chromosome rearrangements in the whole group. Further, bioinformatic analysis in rodents is of limited utility to test evolutionary hypotheses because such comparison are possible between only two murine rodents, mouse (*Mus musculus*) and rat (*Rattus norvegicus*), which were assembled to the chromosomal level. Comparison of genomes exclusively of these two species does not reflect the evolution trends in the whole order Rodentia, which consists of over 30 families.

The rich speciation history of Arvicolinae has long drawn the attention of cytogeneticists to this subfamily. Karyotypes of almost all species were characterized and it is known that Arvicolinae is one of the most karyologically diverse taxonomic groups of mammals with diploid chromosome numbers ranging from 17 to 64. Of special significance are the unusually high number of intraspecific chromosomal polymorphisms which have been described in the subfamily^9,10^ including unusual systems of sex chromosomes and number of species with additional (B) chromosomes^11^. It was suggested that such significant karyotypic variations were the result of so-called catastrophic chromosome evolution that is characterized by ten-fold increase in the rate of chromosome rearrangements compared to average mammalian taxon.

Comparative molecular cytogenetic studies showed that karyotype evolution of Arvicolinae was characterized by multiple interchromosomal rearrangements or translocations^12^. The differences in G-banding pattern suggest that intrachromosomal rearrangements are present within syntenic segments. However, the levels of intrachromosomal rearrangements in Arvicoline cannot be thoroughly investigated unless molecular cytogenetic approaches are used^13^. Up to now the question remains open on whether the rate of intrachromosomal rearrangements is significantly elevated in respect to interchromosomal rearrangements and in general if intrachromosomal rearrangements have an important role in genome evolution and speciation in Arvicolinae.

The major rearrangements between chromosomes in Arvicolinae were thoroughly studied by comparative chromosome painting and the scenario of chromosomal changes was deduced along with karyotype of the ancestor^12,14,15^. The Ancestral Arvicolinae karyotype (AAK)^16^ consists of 27 syntenic blocks. Here we selected three syntenies, which are present as separate autosomes in the majority of studied vole species and in some other arvicolines^12,13,15,17^. The three autosomes have very similar G-banding among studied species but are characterized by a considerable variation of the centromere position. Apparently, the morphology of the conserved syntenic blocks have been altered by intrachromosomal changes, not necessarily limited to centromere reposition events^12,13^. We used microdissection derived regional probes to look for changes within the syntenic blocks. This approach was proven to be productive on a small set of species in our recent, previous pilot study^13^.

Here these three sets were used to analyze 28 Eurasian species presenting an array of 4 arvicoline tribes and 11 genera to estimate the stability of the segment order within these blocks in evolution. Analyses of the distribution pattern of hybridized microdissected painting probes across species uncovered a large number of previously undocumented intrachromosomal rearrangements. Our results appear to support the hypothesis that intrachromosomal rearrangements modulating genome organization have accompanied rapid arvicoline speciation.

## Results

**Figure 1:**
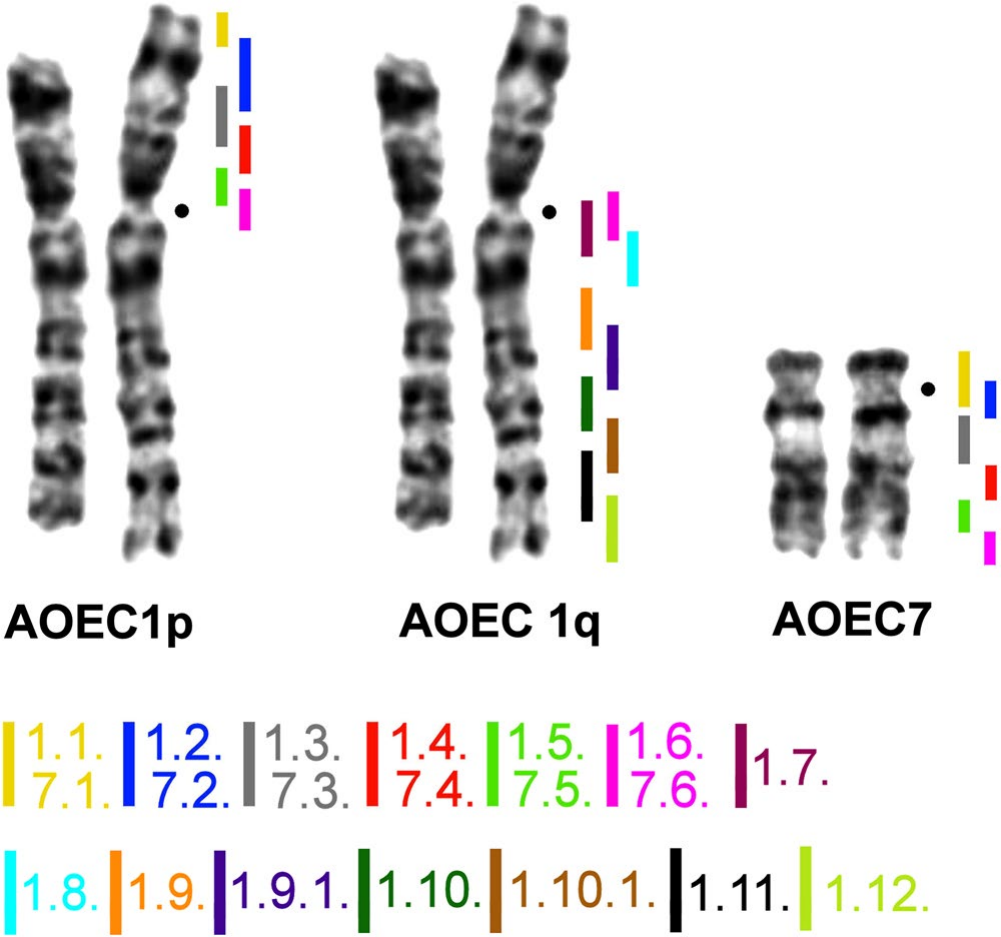
The series of region-specific probes obtained for three ancestral arvicoline syntenic segments (AAK1, 3, and 7) by microdissection of *Alexandromys oeconomus* chromosomes 1 and 7 (G-banded). Color-code of probes and corresponding probe numbers are shown below. Black dots mark the position of centromeres.

We generated three sets of region-specific probes by microdissection of *A*. *oeconomus* (tribe Arvicolini) chromosomes for evolutionary conserved syntenic blocks 1, 3, and 7 of the ancestral Arvicolinae karyotype. The generated sets included region-specific probes from the q-arm of *A*. *oeconomus* (AOEC) chromosome 1 (AOEC1q = AAK1) and whole chromosome 7 (AOEC7 = AAK3). We also employed a set of region-specific probes from the AOEC1p = AAK7, which was described previously^13^. FISH on metaphase chromosomes of *A*. *oeconomus* was performed to map the precise localization of each probe. In total, we obtained 14 partly overlapping probes covering AOEC1 and six probes covering AOEC7 (Fig. 1).

The sets of probes were used for the comparison of chromosomes of the wide range of Arvicolinae species (Table 1).

**Table 1:** List of 28 Arvicolinae species used in the study representing 4 arvicoline tribes and 11 genera.

Tribe	Subtribe	Genus	Subgenus	Species/Subspecies	Abbreviation	2n	Reference to *M*. *agrestis* probe localization
Arvicolini	*Arvicolina*	*Arvicola*		*A*. *amphibius* (=*terrestris*)	AAMP	36	^15^
	*Microtina*	*Alexandromys*		*A*. *evoronensis*	AEVO	36	—
				*A*. *fortis*	AFOR	52	—
				*A*. gromovi**	AGRO	44	—
				*A*. *maximowiczii*	AMAX	44	—
				*A*. *mongolicus*	AMON	50	—
				*A*. *mujanensis*	AMUJ	38	^10^
				*A*. *oeconomus*	AOEC	30	^14^
		*Blanfordimys*		*B*. *afghanus*^*¶*^	BAFG	58	—
				*B*. *juldaschi*^*¶¶*^	BJUL	54	—
		*Chionomys*		*C*. *gud*	CGUD	54	—
		*Lasiopodomys*	*Lasiopodomys*	*L*. *brandtii*	LBRA	34	^16^
			*Stenocranius*	*L*. gregalis*	LGRE	36	^12, 16^
		*Microtus*	*Agricola*	*M*. *agrestis*	MAGR	50	^12^
			*Microtus*	*M*. *arvalis*	MARV	46	^12^
				*M*. *rossiaemeridionalis* (*=levis*)	MROS	54	^12^
			*Sumeriomys*	*M*. *dogramacii*^*¶*^	MDOG	48	^12^
				*M*. *guentheri*^¶^	MGUG	54	^12^
				*M*. *schidlovskii*^*¶*^	MSCH	60	—
				*M*. *socialis*	MSOC	62	^12^
		*Terricola*		*T*. *daghestanicus*	TDAG	54	^12^
				*T*. *majori*	TMAJ	54	—
				*T*. *savii*^*¶*^	TSAV	54	—
Dicrostonychini		*Dicrostonyx*	*Misothermus*	*D*. *torquatus*	DTOR	45 + B	^15^
Ellobiini		*Ellobius*	*Ellobius*	*E*. *talpinus*	ETAL	54	^17, 32^
				*E*. *tancrei*	ETAN	50	—
Myodini		*Alticola*	*Alticola*	*A*. *olchonensis*	AOLC	56	—
		*Myodes* (=*Clethrionomys*)		*M*. (=*C*.) *rutilus*	MRUT	56	^15^

Overall species names here follow the latest checklist “The mammals of Russia: a taxonomic and geographic ref.^47^”, names in brackets are outdated or follow other sources. ^¶^The systematic status of the species defined by^48^. ^¶¶^The systematic status of the species defined by^49^. *Belonged to *Microtus* genus and *Stenocranius* subgenus in^7^. **The species is listed as *M*. *maximowizcii* in^7^. Minus signs indicate that the species have not been involved in comparative studies with painting probes yet or specimens with a different from previously published chromosome number were investigated here.

**Figure 2:**
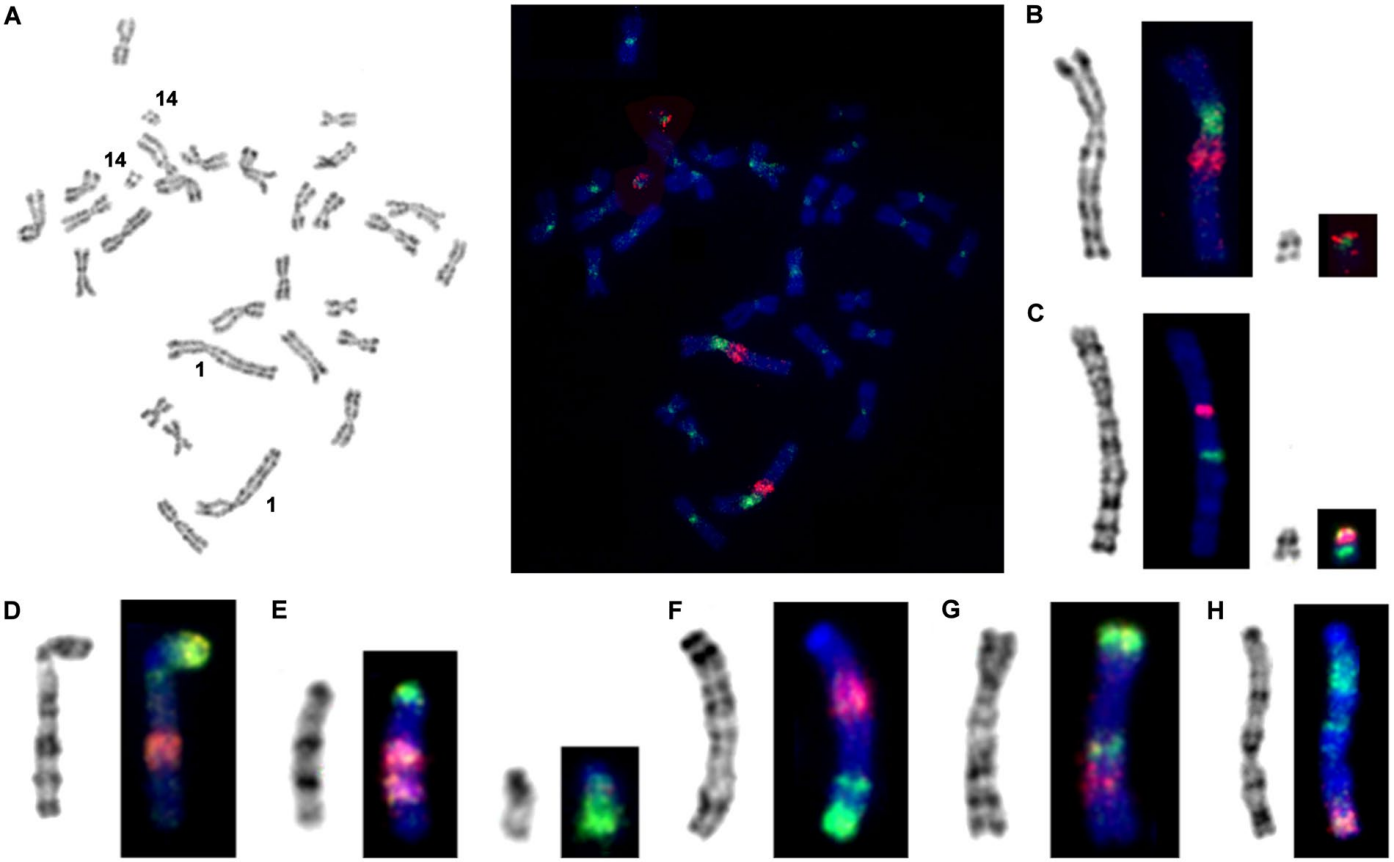
FISH of microdissection-derived painting probes: (**A**) painting probes 1.6. (green) and 1.8. (red) on chromosomes of *A*. *oeconomus*; (**B**) G-banded chromosomes AOEC1 and AOEC14 form (**A**) and the chromosomes after FISH; (**C**) FISH of telomeric DNA probe (red) and rDNA probe (green) onto chromosomes AOEC1 and AOEC14^15^; painting probes 1.7. (green) and 1.10. (red) on chromosomes of *A*. *amphibious* (**D**), *A*. *olchonensis* (**E**), *M*. *schidlovskii* (**F**,**G**) painting probes 1.9.1. (green) and 1.10.1. (red) on chromosome 1 of *A*. *maximowiczii*; (**H**) painting probes 1.9. (green) and 1.12. (red) on chromosome 6 of *M*. *rutilus*. G-banded chromosomes are shown on the left.

We used dual-color FISH with different combinations of probes for precise description of inrtachromosomal rearrangements. The efficacy of probe hybridization differed between species but was sufficiently strong for clear mapping in all cases. Examples of fluorescence *in situ* hybridizations are shown in Fig. 2.

**Figure 3:**
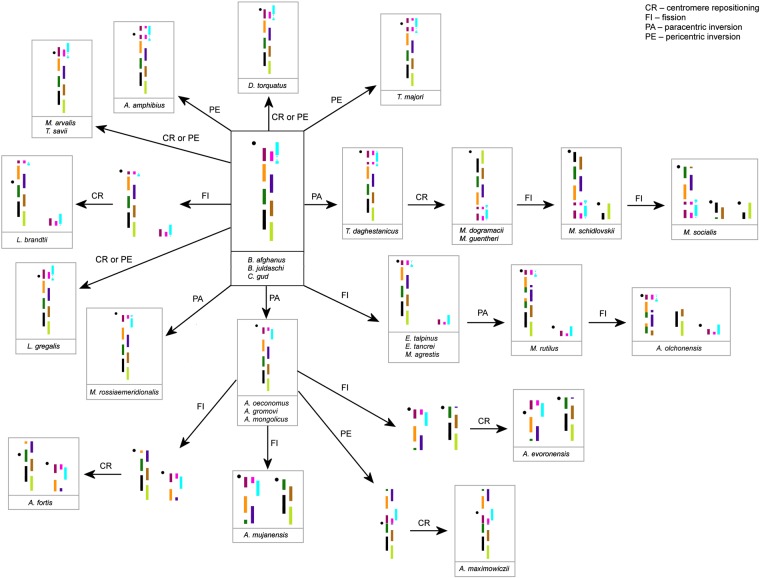
The scheme of evolutionary rearrangements in AAK1 (=AOEC1q) in arvicoline species. The color code corresponds to that in Fig. 1. The ancestral state of AAK1 is shown in the centre in the black frame. Below each color scheme there is a list of species carrying this particular type of chromosome. Black dots indicate centromere position. AAK3 and AAK7 schemes are shown on Supplemental Figs S1 and S2.

After hybridization on *A*. *oeconomus* metaphases, two microdissected probes in addition to a signal on the target chromosome gave additional signals on other chromosomes. Probe 1.6., which covered the pericentromeric region of chromosome 1, additionally hybridized to the pericentromeric regions of all *A*. *oeconomus* chromosomes. Probe 1.8. was obtained from the region of AOEC1, which carries the rRNA genes. In the karyotype of *A*. *oeconomus* in addition to chromosome 1 the probe labeled chromosome 14, which also carries the rRNA genes (Fig. 2A–C). Occasionally both the probes gave slight background signals on chromosomes of other *Alexandromys* species. When analyzing the results to determine the presence and type of intrachromosomal rearrangements, localizations of all probes and their position relative to centromere were taken into account. Notably, in all cases we were able to define the multiple intrachromosomal rearrangements of AAK that gave origin to the chromosomes of extant arvicoline species (Figs 3, S1 and S2, Table 2). The hybridization pattern of the microdissection-derived probes showed that small pericentromeric signals homologous to *Mesocricetus auratus* chromosome 6 were previously undetected on chromosomes MAGR2 and AAMP2q^15^.

**Table 2:** Numbers and types of rearrangements in the ancestral arvicoline chromosomes.

	CR	FI	PA	PE	PE or CR	?
AAK1	5	8	4	3	3	—
AAK3	1	1	6	—	2	1
AAK7	5	2	3	3	—	—

CR - centromere reposition, FI - fission, PA - paracentric inversion, PE - pericentric inversion,? - unclear type of rearrangement.

In certain cases we failed to distinguish pericentromeric inversions and cenrtomere repositions due to limited resolution of the method used (see discussion and)^13^. But we found that centromere repositioning predominated among intrachromosomal rearrangements in syntenies AAK1 and AAK7, while in AAK3 paracentric inversions were more frequent (Table 2).

## Discussion

The conservation of extended chromosome segments in Arvicolinae was demonstrated with whole chromosome-specific probes^12,15,16^. However, our recent comparison of four arvicoline karyotypes with regional microdissection-derived probes revealed a significant number of inversions and centromere shifts inside one conserved region – AAK7^13^. These results raised several intriguing questions. Is the phenomenon of the increased rate of intrachromosomal rearrangements within seemingly evolutionary conserved syntenic regions genome-wide or it occurs only in some local genomic regions? Is such an increased rate arvicoline-constrained or is it a general process characteristic for rodents or even mammals? To answer these questions we have characterized here in greater detail arvicoline conserved regions homologous to AAK1, 3, and 7. We greatly expanded the number of species sampled to 28, representing different arvicoline genera and tribes (Table 1).

**Figure 4:**
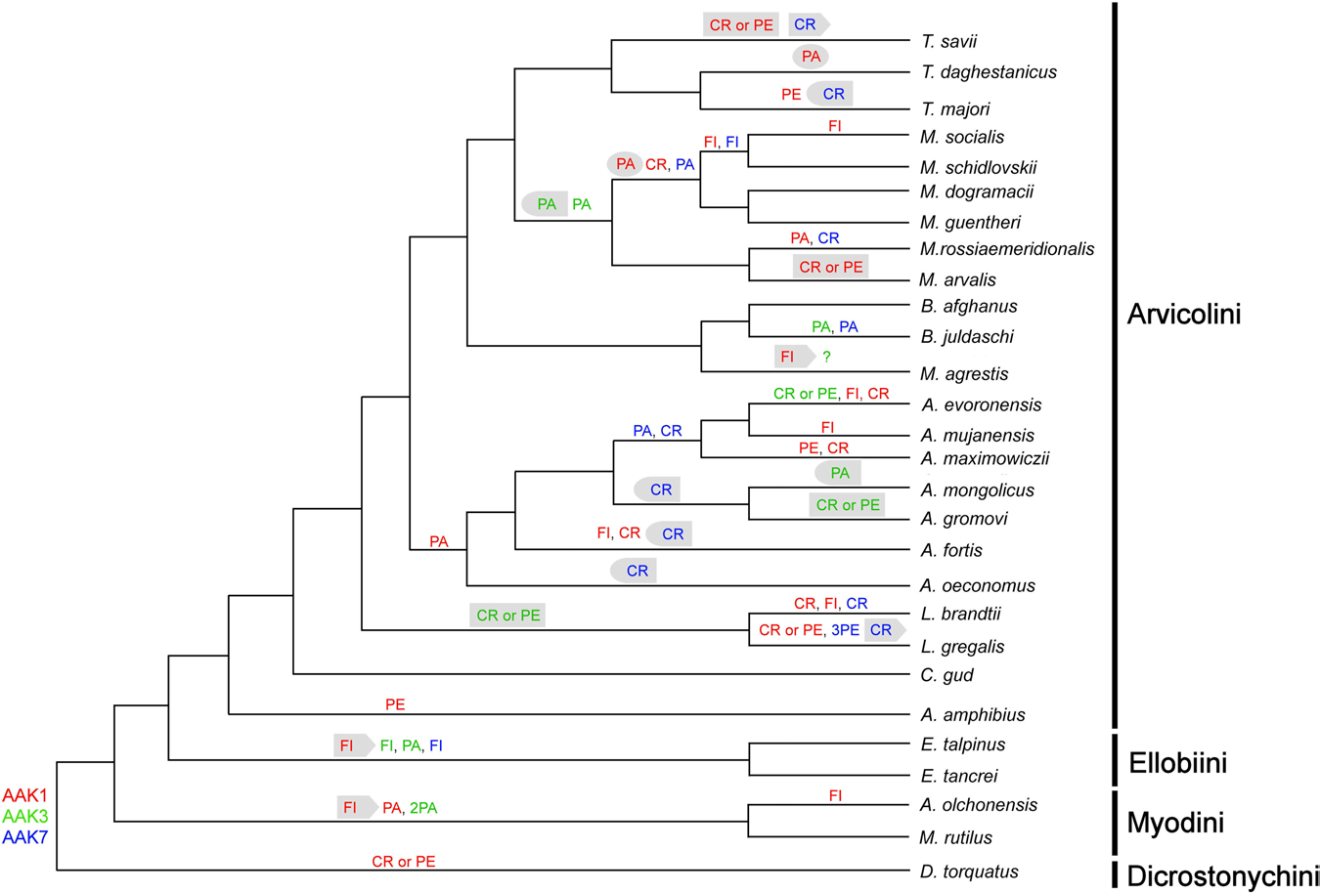
The phylogenetic tree of arvicoline species is based on^2,4,5^. Specific rearrangements are shown above each branch and correspond to AAK1 (red), AAK3 (green), AAK7 (blue). Homoplasies found in different branches were placed in the same-shaped grey figures. The tree only shows the pattern of branching, but not the relative scale of it. The length of the branches is not informative.

AAK was established based on *Microtus agresis* (MAGR) chromosome homologues^12,13,15,17^. AAK1 and AAK3 are homologous to the association of *Microtus agrestis* chromosomes 2/8a and MAGR3, respectively. AAK7 is homologous to the distal part of MAGR1. The most complicated situation was observed in the region homologous to AAK1. Based on the results of comparative chromosome painting, it was previously determined that the chromosome AAK1 is homologous to the regions of chromosomes MAGR2 and 8. Associations MAGR2/8 and 2/8/2 with different centromere positions were revealed in the karyotypes of the investigated voles^12,15,16^. Hybridization of region-specific probes allowed us to correct inaccuracies from earlier chromosome painting data and identify small segments in the pericentromeric regions in MAGR2 and AAMP2q^15^. It was the elimination of these inaccuracies that allowed us to reconstruct the ancestral structure of AAK1 (Figs 3 and 4). In some species (*D*. *torquatus*, *L*. *gregalis*, *M*. *arvalis*, and *T*. *savii*) we could not unambiguously distinguish the type of rearrangement that caused changes in centromere position in the region covered by probes 1.6–1.8. Finer molecular cytogenetic tools will be needed to resolve this question.

Mapping intrachromosomal rearrangements onto a phylogenetic tree clearly identifies the main types of chromosomal changes characteristic not only for taxonomic group, but also for syntenic segments. Consequently, we were able to determine that centromere repositioning predominated in the areas homologous to AAK1 and AAK7 (5 centromere repositioning events against 3–4 para- or pericentric inversions). However, in the AAK3 region, paracentric inversions were more common (6 paracentric inversions against a single documented centromere repositioning) (Figs S1 and S2, Table 2). At the same time, we were able to show that the structure of chromosomes of modern species was formed due to one or two intrachromosomal rearrangements in the chromosomes of the arvicoline ancestor. The possible contribution of paracentric inversions to the evolution of rodent genomes was previously assumed only on the basis of a comparison of the genomes sequences of mouse and rat. Extensive data confirming or refuting this assumption was not collected^11^. On the contrary, a considerable input of pericentric inversions in evolution of some rodent groups (*Mastomys*, *Peromyscus*) was reported^18–20^. There is reason to believe that intrachromosomal rearrangements, at least inside AAK7, are typical for Arvicolinae. AAK7, is homologous to the whole chromosome 9 in the mouse genome^14^. According to the sequencing data, almost the whole of mouse chromosome 9 is homologous to rat chromosome 8, no intrachromosomal rearrangements in this segment were detected^21^. A translocation of a tiny fragment distinguishes mouse chromosome 9 from the homological chromosome 5 of *Microtus ochrogaster*^22^. AAK1 and AAK3 are homologous to different multiple mouse chromosomes that makes it impossible to compare at present the cytogenetic data obtained in our research with the sequencing data. However, here we show that both type of inversions and centromere repositions were fixed in arvicoline genomes. It appears that these intrachromosomal rearrangements have accompanied the rapid speciation of the taxon.

When we placed the intrachromosomal rearrangements on the arvicoline phylogenetic tree we found that some rearrangements were synapomorphic for whole genera or tribes (Fig. 4). Intrachromosomal rearrangements predominated on interchromosomal rearrangements in the three analyzed regions. Only *Ellobius* had more fissions than intrachromosomal rearrangements. In other branches there were up to five times more intra- than interchromosomal rearrangements (Fig. 4). Thus, it can be concluded that in Arvicolinae, and possibly in other rodents and even in some other mammals or vertebrates, intrachromosomal rearrangements were key rearrangements in evolution. Indeed, it could be hypothesized that intrachromosomal rearrangements were the primary driving force of chromosome evolution in mammals. This process was underestimated or has gone unnoticed behind the large-scale rearrangements such as fissions, fusions or large inversions that were pictured by several decades of comparative whole-chromosome studies. Our data indicate that widely used methods of analyses of chromosome evolution such as chromosome painting currently unable to detect or miss large number of intrachromosomal rearrangements. We show that currently only the application of subchromosomal probes can reliably reveal intrachromosomal changes across wide range of species. However, we must still admit that three decades of comparative chromosome painting in over 300 species provided a picture of chromosome evolution across the whole class of Mammalia revealing trends of interchromosomal genomic exchanges^23,24^. However, bioinformatic comparison has yielded only limited view of this same process in less than 20 representative species^25^. Undoubtedly, however, the data obtained provide an excellent basis for future comparison of the genomes of arvicoline and other mammals, when the genomes of a larger number of species will be sequenced and assembled to chromosome level.

Recently it was revealed that interspecific pericentric inversions prevail in closely related bird species with overlapping ranges^26^. In our case, the ranges of most species included in the study overlap considerably^27^. Even between a pair of sibling species with overlapping ranges and similar phenotypes, there are significant karyotype differences and intraspecific chromosome polymorphisms. In spite of the relatively young evolutionary age of the whole subfamily, only few hybrid specimens between *M*. *arvalis* and *M*. *rossiaemeridionalis* were found in nature, restricted to ecologically disturbed areas, but there is no natural hybrid zone^28^. Karyotypes of these species differ significantly in morphology of middle- and small-sized autosomes and X chromosome that were not analyzed here^12,29^. According to previously published data the interspecies difference in structure of the euchromatic part of the X was caused by inversions^29^. We found only one intrachromosomal rearrangement between *M*. *arvalis* and *M*. *rossiaemeridionalis* in AAK1, but after evaluation of the morphology of middle- and small-sized chromosomes we presume that it was changed due to pericentric inversions or centromere repositions. The fact that only few random hybrids between *M*. *arvalis* and *M*. *rossiaemeridionalis* were found on a huge overlapping area supports the hypothesis that these morphologically indistinguishable twin species are reproductively isolated, and perhaps intrachromosomal rearrangements are a contributing factor a phenomena noted in birds^26,30^.

Here we can note that seven species of Far Eastern voles (genus *Alexandromys*) have partial overlapping of ranges in some districts to the east of lake Baikal^27^. Comparisons show that besides a significant number of Robertsonian translocations, para- and pericentric inversions also played a significant role in species isolation (Fig. 4). In addition to the intrachromosomal rearrangements described here, the species have morphologically different X chromosomes. Further, multiple cases of autosomal polymorphism due to pericentric inversions were reported^10,12^. It seems probable that then that karyopytic reorganizations due to intrachromosomal rearrangements also could contribute to the reproductive isolation of species with overlapping ranges.

According to a recently suggested hypothesis, inversions in birds are more frequently fixed in sex chromosomes than in autosomes^26^. Among eutherian mammals, it is generally thought that X-chromosomes are remarkabley conserved. However, there are notable exceptions. For example, the cetartiodactyl X chromosome has incorporated multiple inversions and centromere repositioning in course of evolution. In grey voles (genus *Microtus*) inversions were responsible for interspecies difference in the structure of the euchromatic part of X chromosomes^29,31^. We have yet to test the hypothesis that sex chromosome rearrangements have a faster rate of fixation than those on autosomes. However, the fact that in arvicolines the morphology of sex chromosomes varies considerably could support this hypothesis. Other questions regarding rearrangements inside conserved evolutionary segment remain open. Are certain syntenic blocks less prone to intrachromosomal rearrangements and remain conserved through the evolution? Are these intrasyntenic changes randomly distributed throughout the genome? Here we studied only three conserved autosomal blocks out of 27 in AAK. It is important to mention that among 28 species included in our analysis we did not find any intrachromosomal rearrangements in the three tested regions between some closely related species, such as *Ellobius talpinus/E*. *tancrei* or *M*. *dogramacii/M*. *guentheri*. Moreover, these two pairs of species also have no differences at the cytogenetic level between their X chromosomes^12,32^. These results may indicate that evolution in the taxonomically and karyotypically diverse taxon - Arvicolinae - could occur due to different mechanisms or could diversely affect different conserved syntenies.

In conclusion, we again underline that the comparative contribution of inter- and intrachromosomal rearrangements to genome evolution still remains elusive. However, overall our results lend support to the conclusion that the frequency and importance of intrachromosomal rearrangements may have been significantly underestimated, especially in some species^11,33,34^. Unfortunately, different approaches (bioinformatics, cytogenetics) often provide conflicting results about the number and types of rearrangements. In some taxa, it was shown that intrachromosomal rearrangements are more frequent than interchromosomal rearrangements^31,35,36^. Notably, the contribution of intrachromosomal rearrangements was more significant in rapidly evolving groups. Here we demonstrate that this was also the case for the rapidly evolving voles.

Our results allow us to show that multiple changes took places within syntenic blocks in Arvicolinae karyotypes. In all the regions analyzed here, the number of detected intrachromosomal rearrangements exceeded the number of interchromosomal rearrangements. However, we cannot rule out that different types of rearrangements may prevail in different syntenic blocks. Further, genome evolution in the taxonomically and karyotypically diverse group may also occur due to different mechanisms. Without doubt the complexity of genome evolution will increase as more the use of higher resolution methods (BAC-FISH, sequences comparison) are applied to a wide range of genomes. It is probable given the repeatedly noted contribution of intrachromosomal rearrangements on the formation of reproductive barriers between populations^11,37^, that such rearrangements also play a significant role in the processes of rapid speciation in Arvicolinae.

## Methods

### Compliance with ethical standards

All applicable international, national, and/or institutional guidelines for the care and use of animals were followed. All experiments were approved by the Ethics Committee on Animal and Human Research of the Institute of Molecular and Cellular Biology, Siberian Branch of the Russian Academy of Sciences, Russia (order No. 32 of May 5, 2017). This article does not contain any studies with human participants performed by any of the authors.

### Species sampled

We used chromosome suspensions obtained from cell lines in the Laboratory of Animal Cytogenetics, the IMCB SB RAS, Russia. All cell lines were retrieved from the IMCB SB RAS cell bank (“The general collection of cell cultures”, № 0310-2016-0002). The list of species is presented in the Table 1: the origin of each sample, establishment of cell lines, karyotype description for each studied species were previously reported^12,14,15^.

### Chromosome preparation and chromosome staining

Chromosome suspensions were obtained from cell lines according to earlier published protocols^38,39^. G-banding was performed on chromosomes of all species prior to FISH using the standard trypsin/Giemsa treatment procedure^40^.

### Microdissection, probe amplification and labeling

Microdissection was performed as described earlier^41^. Ten copies of each region were collected. Chromosomal DNA was amplified and labeled using WGA kits (Sigma). Region-specific painting probes covering the whole p-arm of the *A*. *oeconomus* chromosome 1 were described earlier^13^. In total we obtained 20 region-specific painting probes from chromosomes 1 and 7 of *A*. *oeconomus* (Fig. 1). The telomeric DNA probe was generated by PCR using the oligonucleotides (TTAGGG)_5_ and (CCCTAA)_5_^42^. Clones of human ribosomal DNA containing partial 18S, full 5.8S, part of the 28S ribosomal genes, and two internal transcribed spacers were obtained as described in^43^.

### Fluorescence *in situ* hybridization (FISH)

The painting probes were labeled with either biotin or digoxigenin by DOP-PCR amplification as described previously^14,15,44^. FISH was performed following previously published protocols^45,46^. Images were captured using VideoTest-FISH software (Zenit) with a JenOptic CCD camera mounted on an Olympus BX53 microscope. Hybridization signals were assigned to specific chromosome regions defined by G-banding pattern captured by the CCD camera prior to FISH. All images were processed using Corel Paint Shop Pro X2 (Corel).

## Electronic supplementary material

Figures S1 and S2
