# Supplementary material for: Multiple intrasyntenic rearrangements and rapid speciation in voles
Source: Sci Rep. 2018 Oct 8;8:14980. doi: 10.1038/s41598-018-33300-6 (PMC6175948; doi:10.1038/s41598-018-33300-6)
Supplement: Supplementary file 1 — Figures S1 and S2 [file 41598_2018_33300_MOESM1_ESM.doc]

**Multiple intrasyntenic rearrangements and rapid speciation in voles**

Svetlana A. Romanenko^1,2*^, Natalya A. Serdyukova^1^, Polina L. Perelman^1,2^, Vladimir A. Trifonov^1,2^, Feodor N. Golenishchev^3^, Nina Sh. Bulatova^4^, Roscoe Stanyon^5^, Alexander S. Graphodatsky^1,2^


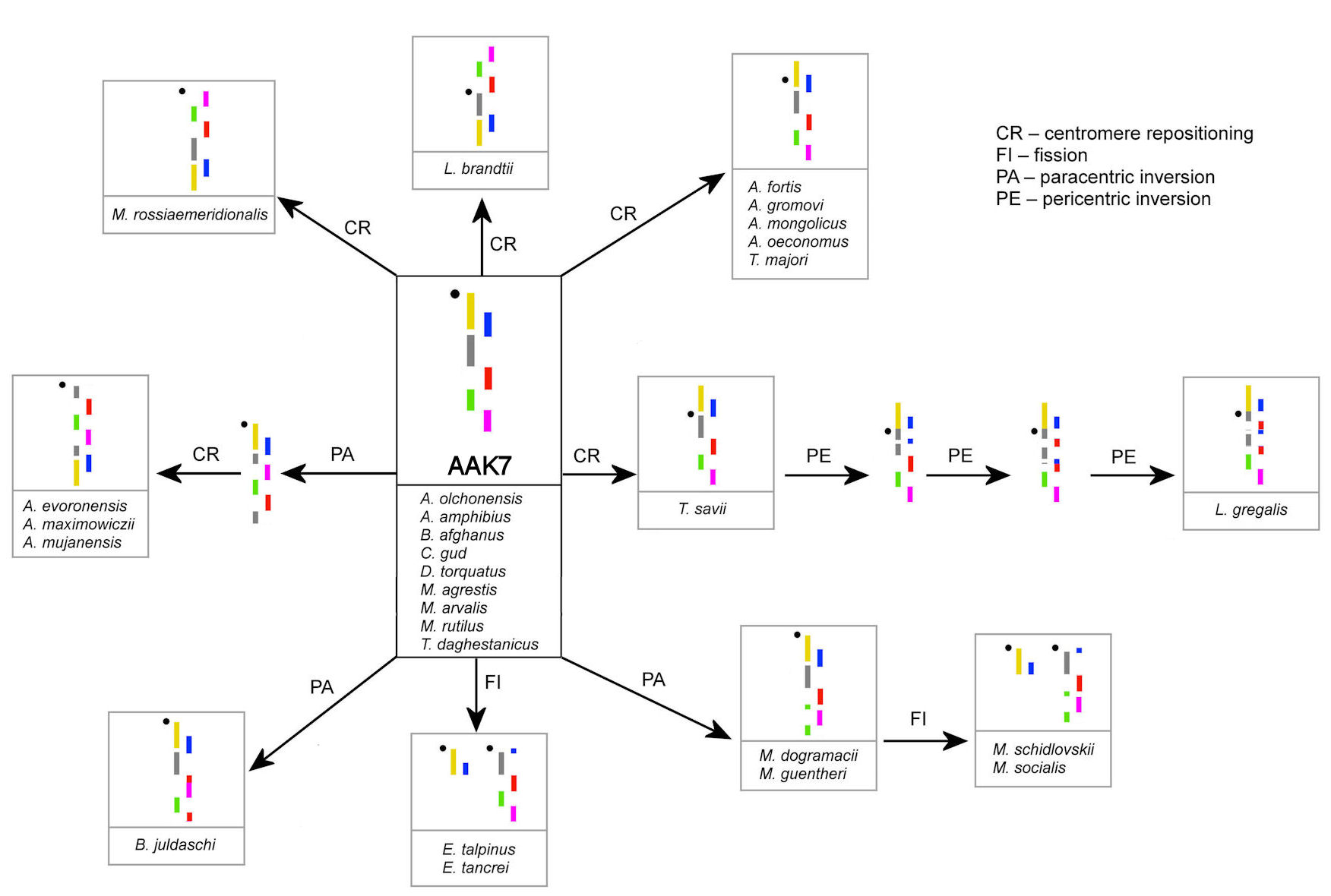


**Figure S1.** The scheme of rearrangements in AAK7 (=AOEC1p) in arvicoline species. The color code corresponds to that in Figure 1. The ancestral state of AAK7 is shown in the central black frame. Below the color scheme there is a list of species having this type of chromosome. Black dot marks position of centromere.


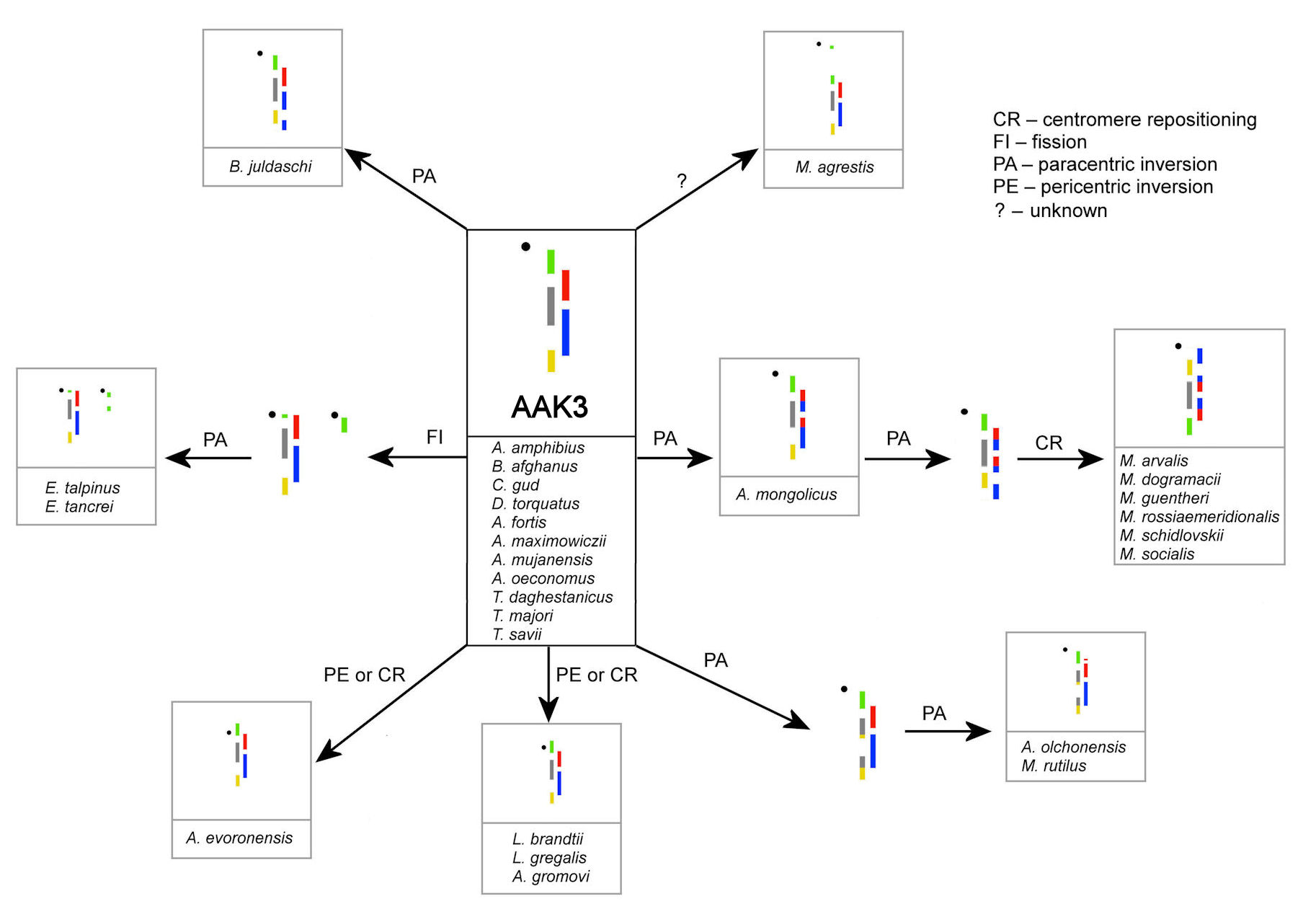


**Figure S2.** The scheme of rearrangements in AAK3 (=AOEC7) in arvicoline species. The color code corresponds to that in Figure 1. The ancestral state of AAK3 is shown in the centre in the black frame. Below the color scheme there is a list of species having this type of chromosome. Black dots marked the positions of centromeres.
